# Subdividing the Digital Divide:  Differences in Internet Access and Use among Rural Residents with Medical Limitations

**DOI:** 10.2196/jmir.1534

**Published:** 2011-03-03

**Authors:** Jong-Yi Wang, Kevin Bennett, Janice Probst

**Affiliations:** ^3^Department of Health Services Policy and ManagementArnold School of Public HealthUniversity of South CarolinaColumbia, SCUnited States; ^2^Department of Family & Preventive MedicineSchool of MedicineUniversity of South CarolinaColumbia, SCUnited States; ^1^Department and Graduate Institute of Health Services AdministrationChina Medical UniversityNo.91 Hsueh-Shih RoadTaichungTaiwan

**Keywords:** Disabled Persons, Medical Condition Limiting Travel, Internet, Rural Communities, Minority Health

## Abstract

**Background:**

Access to health care is often contingent upon an individual’s ability to travel for services. Certain groups, such as those with physical limitations and rural residents, have more travel barriers than other groups, reducing their access to services. The use of the Internet may be a way for these groups to seek care or information to support their health care needs.

**Objective:**

The purpose of this study was to examine Internet use among those whose are, for medical reasons, limited in their ability to travel. We also examined disparities in Internet use by race/ethnicity and rural residence, particularly among persons with medical conditions.

**Methods:**

We used data from the 2001 National Household Travel Survey (NHTS), a nationally representative sample of US households, to examine Internet use among individuals with medical conditions, rural residents, and minority populations. Internet use was defined as any use within the past 6 months; among users, frequency of use and location of use were explored. Control variables included sociodemographics, family life cycle, employment status, region, and job density in the community. All analyses were weighted to reflect the complex NHTS sampling frame.

**Results:**

Individuals with medical conditions were far less likely to report Internet use than those without medical conditions (32.6% vs 70.3%, *P* < .001). Similarly, rural residents were less likely to report Internet access and use than urban residents (59.7% vs 69.4%, *P* < .001). Nationally, 72.8% of white respondents, versus 65.7% of persons of “other” race, 51.5% of African Americans, and 38.0% of Hispanics reported accessing the Internet (*P* < .001). In adjusted analyses, persons with medical conditions and minority populations were less likely to report Internet use. Rural-urban differences were no longer significant with demographic and ecological characteristics held constant.

**Conclusions:**

This analysis confirmed previous findings of a digital divide between urban and rural residents. Internet use and frequency was also lower among those reporting a medical condition than among those without a condition. After we controlled for many factors, however, African Americans and Hispanics were still less likely to use the Internet, and to use it less often, than whites. Policy makers should look for ways to improve the access to, and use of, the Internet among these populations.

## Introduction

A substantial number of Americans have physical or other conditions that reduce their ability to travel. Such conditions hamper their ability to see, operate a vehicle, gain access to public transportation, or walk to a desired destination. Many of these individuals, therefore, rely upon family members, friends, or other modes of transportation for their travel needs [1].

Rural residents have a slightly higher rate of disabling conditions than urban residents, particularly in the South [2]. These rural residents are especially vulnerable in regard to travel restrictions. The reduced availability of services, and relatively greater distance between services and housing centers, and the reduced availability of public transportation exacerbate these resident’s travel difficulties [3]. These barriers in available transportation can lead to reduced utilization of services [1]. 

The evolution of the Internet as a resource, especially for health care information and services, may be an ameliorant for those with travel difficulties. Many patients rely on the Internet for gathering information about their conditions and treatment options, and for communication with their providers. Patients also use the Internet to garner social support, using the interface as a coping mechanism [4-6]. The Internet also can play an important role in the education and recruitment of patients for specific services or programs [7,8].

Internet access is influenced by available telecommunication infrastructure and the affordability of Internet services [9]. The high cost of providing services across the more widely dispersed rural population is one barrier to the development of infrastructure in rural areas [10]. As a result, rural areas lag behind in the infrastructure required for optimal Internet use (such as broadband or other high-speed service), and rural residents have lower reported use of the Internet than urban residents [11]. Since home availability of the Internet remains low in rural communities, and usage at work was also lower [12], rural residents were more likely than those in urban or suburban areas to use a source other than work or home for accessing the Internet [13].

Sociodemographic characteristics are also significantly associated with Internet use. African Americans and Hispanics were less likely than whites to report Internet access, and Hispanics were less likely than whites to report using the Internet for health-related issues [14]. Other socioeconomic characteristics, such as higher educational levels, younger age, and greater household income, were found to be associated with any prior use of the Internet among surgery patients [7,13]. A Pew Internet surveys found that Internet users who were female, were older, had a higher education and income, were white, were not employed full time, were married, and had a child under 18 living at home were more likely to report using the Internet to search for health information [15].

The digital divide between urban and rural populations has important implications for the health of rural residents, particularly those who are limited in their ability to travel. These individuals, as well as rural populations, generally have reduced access to primary care, coupled with greater travel distances to care [16,17]. They could benefit from Internet access, as Internet availability could facilitate research into health conditions, as well as providing additional links to services. The purpose of this study, therefore, was to examine Internet use among people with limited ability to travel. We also examined disparities in Internet use by race/ethnicity and rural residence, particularly among those with medical conditions.

## Methods

### Data Source

We analyzed a data set not generally used for health services research, the 2001 National Household Travel Survey (NHTS) of the US Department of Transportation. The 2001 NHTS, a multistage telephone interview, obtained information from a nationally representative sample of households from March 2001 through May 2002. Eligible participants were civilian, noninstitutionalized persons who considered themselves primary residents of the households sampled. In addition to examining travel and ability to travel, the 2001 NHTS asked respondents about their Internet use.

The overall response rate for the NHTS was 41% [18]. Survey responses were weighted to account for underresponse among specific populations. After merging the person and household data sets in the 2001 NHTS, we identified 44,507 respondents living in 25,616 households, which represent a weighted population of 200,257,143.

### Definition of Variables

#### Dependent Variables

We defined three dependent variables: whether a respondent had accessed the Internet in the past 6 months (yes/no), frequency of use in the last 6 months among persons who reported use, and location of use among persons who reported use. Frequency of Internet use was measured dichotomously: *frequent use* included “almost every day” or “several times a week,” while *infrequent use* included “once a week” or “once a month”. *Location of use* was characterized by the NHTS as “home only,” “work only,” “other only,” “home and work,” “home and other,” “work and other,” and “home, work, and other.” In multivariate analysis, we compared “home only” to all other categories.

#### Independent Variables

We sought to examine three aspects of a potential digital divide: presence or absence of a *medical condition limiting travel* (hereafter, “medical condition”), *residence*, and *race/ethnicity*. *Medical condition* was coded as “yes” if the respondent indicated that he or she had a medical condition with any of the following characteristics: limits driving to daytime, limits use of public transportation, results in asking for rides, requires giving up driving, requires special transport, and results in less travel. Otherwise, the medical condition variable was coded as “no.” No finer distinctions, such as categories of physical or mental disease, were made available by the survey instrument.

We used the definition of rural used by the 2001 NHTS, developed by Claritas Inc. [18]. This approach divides the United States into grids, with population density within each geographic grid expressed as centiles (0 through 99). The definition of rural included centiles 0 through 19, while centiles 20 and above were considered urban.

Race and ethnicity were coded as white, African American, Hispanic, and other. Persons in multiple race/ethnicity groups were included in the “other” race and ethnicity category.

#### Control Variables

Other factors, in addition to residence and race/ethnicity, are known to influence Internet access and usage. These control variables, held constant in multivariate analysis, were conceptualized into two categories: demographic factors and ecological factors. Demographic factors were the respondent’s age group (<26, 26-50, 51-75, and >75 years), sex, education (high school or lower, college, and graduate school), household income (<$20,000, $20,000-$44,999, $45,000-$70,000, and >$70,000), family life cycle stage (young adult, young family, older family, or retired), and occupation type (sales, clerical, blue collar, white collar, or technical). Ecological factors were region (Northeast, Midwest, South, and West) and job density within the respondent’s area of residence. Job density was defined in the NHTS as “Jobs per square mile - Tract level”. Based on the distribution of job density, we categorized it into three groups: low (fewer than 96.1 jobs per square mile), medium (between 96.1 and 692.3), and high (greater than 692.3).

### Statistical Approach

We first used univariate analysis to describe the study population. We next used bivariate analysis, with Wald chi-square tests of differences, to examine Internet use by the variables of interest (medical conditions, residence, and race/ethnicity). Finally, we conducted multivariate logistic regression to determine whether medical conditions, residence, and race/ethnicity were significantly associated with Internet use when holding demographic and ecological factors equal. All analyses were conducted in SAS-callable SUDAAN version 10 (RTI International, Research Triangle Park, NC, USA) to account for the complex NHTS sampling design. All analyses employed sampling weights, reflecting the underrepresented or oversampled groups in specific states. All testing was two sided and conducted at alpha = .05.

## Results

In 2001, about two-thirds of Americans reported having accessed the Internet within the past 6 months (Table 1). Rural residents were less likely than their urban peers to report accessing the Internet (59.7 versus 69.4%, *P* < .001). Only about a third of persons who reported a medical condition that impaired their driving (32.6%) reported accessing the Internet, compared to 70.3% among those without a medically limiting condition (*P* < .001). A marked difference was also present across race/ethnicity. Nationally, 72.8% of white respondents, versus 65.7% of persons of “other” race, 51.5% of African Americans, and 38.0% of Hispanics, reported accessing the Internet (*P* < .001). Less than a third of rural African American or Hispanic respondents reported accessing the Internet compared to 64.5% of rural whites (*P* < .001, data not in table).

As might be expected, the likelihood of accessing the Internet increased linearly with education and income, and decreased with age (*P* < .001). Occupational differences may reflect job requirements; individuals in manufacturing and related industries were markedly less likely to report accessing the Internet than were those in other occupations (*P* < .001).

**Table 1:** Reported Internet use within the past 6 months, NHTS 2001^a^, by respondent characteristics (n = 44,507 observations; estimated population 200,257,143)

Percentage reporting Internet use		Unweighted observations	Estimated population	Weighted proportions (%)
Total		30,128	135,011,405	67.4
**Travel limitation due to a medical condition**^b^				
	Yes	1248	5,038,139	32.6
	No	28,880	129,973,266	70.3
**Residence**^b^				
	Rural	6139	23,975,873	59.7
	Urban	23,989	111,035,532	69.4
**Race**^b^				
	White	25,630	103,924,340	72.8
	African American	1336	11,907,786	51.5
	Hispanic	583	4,686,522	38.0
	Other	2579	14,492,757	65.7
**Age group(years)**^b^				
	<26	4734	27,894,654	80.6
	26-50	16,503	77,161,873	77.6
	51-75	8372	28,200,505	52.1
	>75	519	1,754,372	14.6
**Sex**^b^				
	Male	14,325	66,648,850	69.4
	Female	15,803	68,362,555	65.6
**Education**^b^				
	High school or lower	8123	37,041,567	47.0
	College	16,227	72,802,951	79.8
	Graduate school	5223	22,272,126	88.2
	Not ascertained	555	2,894,760	58.9
**Household income**^b^				
	<$20,000	2059	11,229,371	33.5
	$20,000-$44,999	7109	33,725,334	59.0
	$45,000-$70,000	8158	36,117,045	79.3
	>$70,000	11,254	47,388,158	93.0
	Not ascertained	1548	6,551,497	50.3
**Family life cycle**^b^				
	≥1 adults, no children	10,162	44,707,004	73.3
	≥1 adults, youngest child 0-15	12,381	59,678,984	76.4
	≥1 adults, youngest child 16-21	3382	16,027,166	79.5
	≥1 adults, retired, no children	4203	14,598,251	35.5

**Occupation**^b^				
	Sales or service	5635	26,652,524	73.2
	Clerical or administrative support	2942	13,083,350	85.0
	Manufacturing, construction, maintenance, farming	3051	14,462,202	55.8
	Professional, managerial, or technical	10,419	46,326,247	90.6
	Other	8081	34,487,082	48.3
**Region**^b^				
	Northeast	5799	26,046,879	68.2
	Midwest	7815	31,351,034	68.7
	South	9535	46,769,938	65.0
	West	6979	30,843,554	69.4
**Job density**^b^				
	Low	6763	26,250,968	60.4
	Medium	7766	32,642,536	71.4
	High	15,599	76,117,901	68.5

^a^ NHTS: National Household Travel Survey.

^b^ Between-group differences significant, *P* < .001.

Among persons who did report accessing the Internet, the majority used it daily (54.2%; Table 2). Among persons with medical conditions, more than two-thirds (68.3%) reported accessing the Internet only from home, versus 38.7% of other individuals (*P* < .001). Rural residents were less likely to report daily use (47.0% vs 55.7%), and more likely to report use only once per month (13.3% vs 9.4%), than their urban peers (*P* < .001). Frequency of Internet use differed by race/ethnicity as well (*P* < .001): African Americans and Hispanics were less likely to report almost daily Internet use, and were more likely to report use only once per month. African Americans were less likely to have access at home (34.1%) than either Whites (40.8%) or Hispanics (40.3%), but were more likely to report use at work (9.9%, *P* < .001).

**Table 2:** Frequency and location of use among persons with Internet access, by residence and presence of a medical condition limiting travel

			Medical limitations			Residence			Race/ethnicity				
		All	Limited travel	No limitations	*P*-value	Rural	Urban	*P*-value	White	Afr. Am.^a^	Hispanic	Other	*P*-value
**Frequency of access**					.02			<.001					<.001
	Almost every day	54.2	51.0	54.3		47.0	55.7		55.9	42.5	39.7	56.3	
	Several times a week	23.4	24.3	23.3		25.1	23.0		22.9	28.1	26.2	22.1	
	Once a week	12.3	11.5	12.4		14.6	11.9		11.8	15.4	17.2	11.8	
	Once a month	10.1	13.2	10.0		13.3	9.4		9.4	13.9	16.9	9.8	
**Location of access**					<.001			<.001					<.001
	Home only	39.8	68.3	38.7		44.7	38.8		40.8	34.1	40.3	37.3	
	Work only	7.6	4.1	7.8		8.5	7.4		7.6	9.9	7.5	6.2	
	Home and work	30.7	11.3	31.4		25.1	31.9		31.6	24.2	25.0	31.1	
	Other	21.9	16.3	22.1		21.7	21.9		20.0	31.8	27.2	25.4	

^a^ Afr. Am.: African American.

Adjusted odds for accessing the Internet and factors associated with intensity and location of use among persons who reported Internet access are presented in Table 3. With all personal and ecological characteristics held equal, rural residents were no less likely than urban residents to report accessing the Internet (odds ratio [OR] 0.89, 95% CI 0.76-1.04), and did not differ with regard to frequency or location of use. Among persons with a medical condition that limited travel, the odds of accessing the Internet were lower, even controlling for age and life cycle stage (OR 0.66, 95% CI 0.59-0.74). Medically impaired persons who did access the Internet were most likely to use it at home (OR 1.70, 95% CI 1.43-2.03).

The digital divide between the races in 2001 was extensive (Table 3). All minorities were less likely than whites to report any Internet access (OR 0.38, 95% CI 0.33-0.43 for African American; OR 0.20, 95% CI 0.17-0.24 for Hispanic; OR 0.51, 95% CI 0.45-0.58 for other). For African Americans, the odds of any use, of frequent versus infrequent use, and of use at home versus at other locations were all lower than for whites. Hispanics were similarly less likely to report any use and to report frequent use, although they did not differ in location of use from white respondents.

Other characteristics influenced accessing the Internet and type of use in a manner paralleling the findings shown in Table 1. In adjusted analysis, the odds of reporting any Internet access increased as education or income increased, and decreased as age increased. Women were less likely to report any Internet use and frequent use, with women who did use the Internet being more likely to access it at home than in other locations. Among persons using the Internet, lower income and education were associated with use at home versus other locations.

**Table 3:** Adjusted odds ratios (OR) that an individual will report selected types of Internet use, NHTS 2001^a^

		Internet access		Among respondents using the Internet			
		Within past 6 months		Frequent versus infrequent use		Home versus other location	
		OR	95% CI	OR	95% CI	OR	95% CI
**Travel-limiting medical condition (referent: no condition)**							
	Yes	0.66	0.59-0.74	1.05	0.90-1.22	1.70	1.43-2.03
**Residence (referent: urban)**							
	Rural	0.89	0.76-1.04	0.92	0.81-1.05	1.08	0.93-1.25
**Race/ethnicity (referent: white**)							
	African American	0.38	0.33-0.43	0.67	0.59-0.77	0.76	0.64-0.89
	Hispanic	0.20	0.17-0.24	0.61	0.51-0.74	0.92	0.74-1.14
	Other	0.51	0.45-0.58	0.96	0.84-1.10	0.90	0.81-1.00
**Demographic characteristics**							
**Age group (referent: <26 years)**							
	26-50	0.47	0.41-0.53	0.94	0.86-1.04	1.88	1.73-2.05
	51-75	0.19	0.16-0.21	0.79	0.71-0.88	2.60	2.32-2.92
	>75	0.05	0.04-0.06	0.76	0.56-1.03	4.39	3.25-5.91
**Sex (referent: male)**							
	Female	0.87	0.82-0.93	0.69	0.64-0.74	1.50	1.42-1.60
**Education (referent: graduate school)**							
	High school or lower	0.24	0.20-0.28	0.53	0.48-0.60	1.95	1.73-2.20
	College	0.65	0.56-0.76	0.76	0.68-0.85	1.47	1.33-1.62
	Not ascertained (not interpretable; used to prevent loss of observations)	0.29	0.23-0.38	0.65	0.50-0.85	1.47	1.17-1.86
**Household income (referent: >$70,000)**							
	<$20,000	0.11	0.10-0.13	0.65	0.56-0.75	0.99	0.87-1.13
	$20,000-$44,999	0.24	0.21-0.27	0.68	0.62-0.75	1.44	1.32-1.58
	$45,000-$70,000	0.44	0.38-0.50	0.73	0.67-0.79	1.41	1.30-1.52
	Not ascertained (not interpretable; used to prevent loss of observations)	0.20	0.17-0.23	0.74	0.63-0.89	1.29	1.09-1.53
**Family life cycle (referent:≥1 adults, youngest child 16-21)**							
	≥1 adults, no children	0.81	0.70-0.93	1.22	1.07-1.38	0.68	0.61-0.77
	≥1 adults, youngest child 0-15	0.97	0.83-1.14	0.84	0.75-0.94	1.03	0.92-1.15
	≥1 adults, retired, no children	0.50	0.42-0.59	1.16	0.99-1.36	1.57	1.33-1.85
**Occupation (referent: professional, managerial, or technical)**							
	Sales or service	0.45	0.39-0.51	0.66	0.60-0.73	2.88	2.61-3.16
	Clerical or administrative support	1.09	0.93-1.28	1.00	0.87-1.15	0.89	0.79-1.01
	Manufacturing, construction, maintenance, or farming	0.26	0.23-0.29	0.39	0.35-0.44	5.46	4.92-6.06
	Other	0.36	0.33-0.40	0.77	0.70-0.84	5.49	5.03-5.99

**Ecological factors**							
**Region (referent: West)**							
	Northeast	0.86	0.76-0.98	1.07	0.96-1.18	1.23	1.11-1.36
	Midwest	0.93	0.82-1.05	0.91	0.82-1.00	1.04	0.95-1.15
	South	0.95	0.86-1.06	1.05	0.96-1.15	0.99	0.91-1.07
**Job density (referent: high)**							
	Low	0.89	0.76-1.04	0.88	0.77-1.01	0.88	0.76-1.02
	Medium	1.12	1.02-1.23	0.89	0.82-0.96	1.01	0.93-1.09

^a^ NHTS: National Household Travel Survey.

## Discussion

The present analysis sought to investigate differences in Internet access and use among persons affected by medical conditions, among rural residents, and across racial/ethnic groups. Persons with a medical condition that limits their availability to travel were deemed to have a particular need for Internet access, to allow them to obtain information and social support [4-8]. We found, however, that Internet use and frequency were lower among persons with a medical condition than those without; persons with a medical condition were more likely to access the Internet only from home. Lower odds for any Internet use within the past 6 months and Internet use exclusively from home persisted in adjusted analysis. Other factors not captured by the present analysis, including personal preferences and/or the inability to use computers due to the person’s limitations, may account for this particular type of digital divide.

Further research is needed to explore barriers to Internet use among persons whose travel is limited by medical conditions. Such research must take into consideration that Internet access alone does not always translate into its use for health information and support. Previous research suggests that the proportion of patients with Internet access who use the Internet for health information ranges from 89% among bariatric surgery patients to less than 50% among primary or tertiary care settings [4,19-22]. Thus, efforts should continue not only to improve Internet access among persons with medical conditions, but also to encourage their use of health-related information resources.

The unadjusted findings of the present study supported previous evidence of a geographic digital divide, as rural residents were less likely to use the Internet than their urban counterparts. Adjusted analysis, however, suggested that the characteristics of rural populations, rather than lower technology penetration in rural areas [11], accounts for the differences. With demographic and ecological conditions held constant, rural residents did not differ from their urban peers. In particular, the job-related factors included in the model (job density and occupational type) may explain the rural-urban differences found in the unadjusted analysis. Rural residents were more likely to be in low job density areas and to work in nonwhite-collar occupations, both of which were associated with a reduced likelihood of Internet use [12,13]. This is further supported by rural residents’ report of higher Internet use at home only (44.7%) than urban residents (38.8%).

Our study also confirmed previous work suggesting lower Internet use among African American and Hispanic populations [14]. In both unadjusted and adjusted analyses, all minority groups were less likely than whites to report Internet access within the past 6 months. Disparities in frequent use and use at home persisted among African American and Hispanic respondents even after statistical adjustment for income, education, occupation, and other demographic characteristics. Further research is needed to determine whether these disparities, measured in 2001-2002, persist 8 years later. Should this be the case, additional research will need to explore whether minority populations perceive Internet access to be of lesser utility than do white populations, or experience other cultural barriers to use.

Our study has several limitations. First, the NHTS was not designed for health research; thus, using it to define medically limited individuals may lead to overestimation of those who may be clinically disabled. In addition, all data are based on respondent self-report, which may bias findings in an unknown direction. On the other hand, the NHTS was the only source for information on both travel limitations and Internet use from a random sample of the US population. A second limitation is that the NHTS defines rural differently from many traditional geographic analyses; however, the use of deciles closely mirrors alternative measures, providing a suitable proxy for rurality. The age of the data (2001-2002) may reduce the generalizability of the findings given the rate of technological advancement; future analyses will use newer data as it comes available. Finally, this survey did not inquire about what types of information the user was seeking while accessing the Internet. It would be helpful to know, for example, whether those who have a medical condition that limits travel are seeking health information on the Internet at a rate that differs from those who are not limited.

Despite the limitations, the findings of the present analysis remain important and relevant: the digital divide persists for several vulnerable populations. While it is posited that Internet access can make health expertise broadly available, persons with medical conditions that limit travel, who might benefit from such access, were less likely to use the Internet than their peers. African American and Hispanics also were affected by the digital divide. For rural residents, multivariate analysis suggests that personal characteristics, rather than geography, limit Internet access and use.

## Abbreviations

NHTS: National Household Travel Survey
OR: odds ratio
